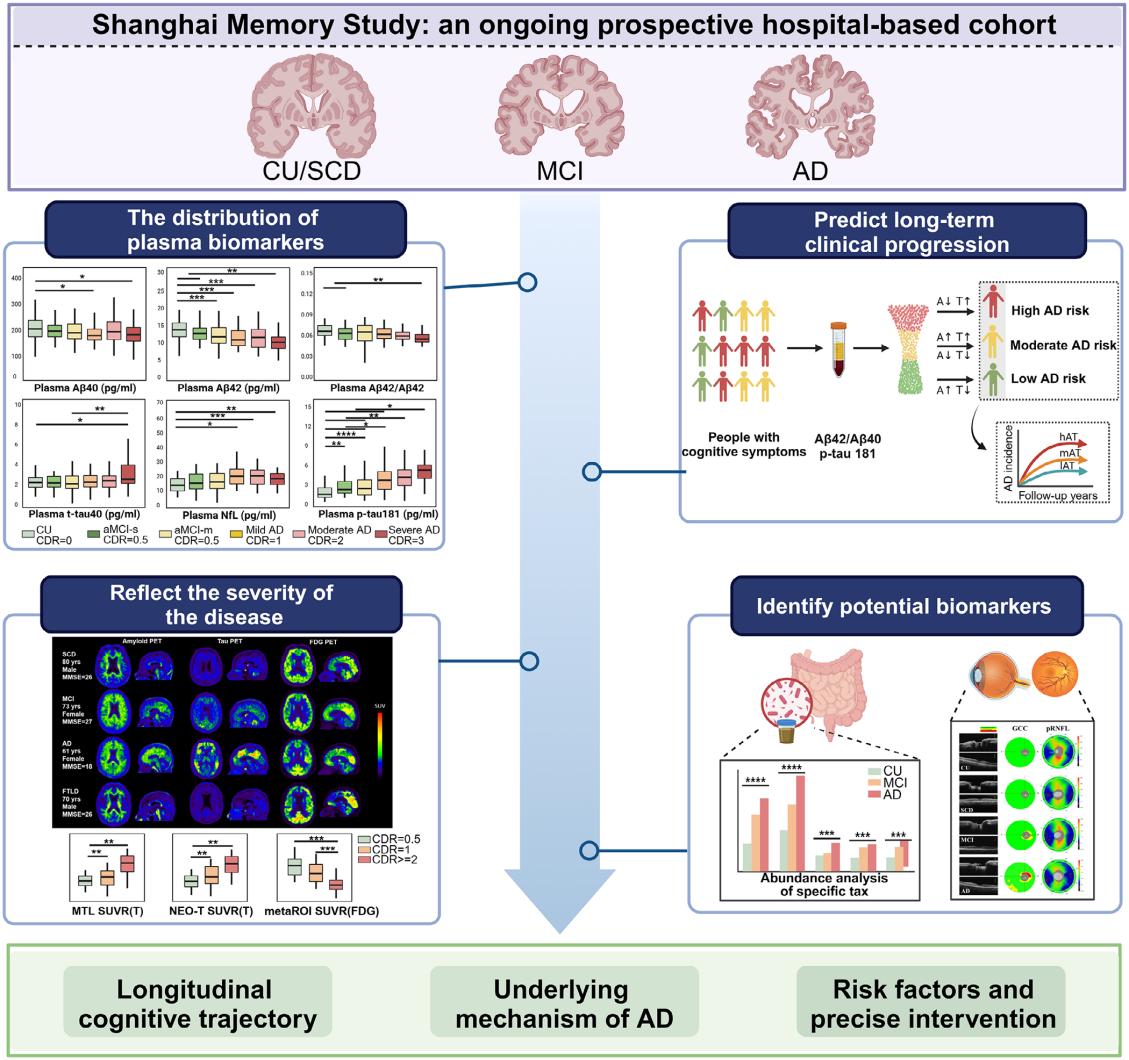# Clinical features, biomarker profile and neuroimaging characteristics in patients from memory clinic in China: The Shanghai Memory Study

**DOI:** 10.1002/alz70856_099230

**Published:** 2025-12-24

**Authors:** Jie Wang, Xiaoxi Ma, Jiaying Lu, Jie Wu, Zhenxu Xiao, Huiwei Zhang, Weiqi Bao, Saineng Ding, Li Zheng, Xiaoniu Liang, Yihui Guan, Chuantao Zuo, Ding Ding, Qianhua Zhao, Shanghai Memory Study Group

**Affiliations:** ^1^ Institute and department of Neurology, Huashan Hospital, Fudan University, Shanghai, China; ^2^ Department of Nuclear Medicine and PET Center, Huashan Hospital, Fudan University, Shanghai, Shanghai, China; ^3^ Department of Nuclear Medicine and PET Center, Huashan Hospital, Fudan University, Shanghai, China; ^4^ National Center for Neurological Disorders, Huashan Hospital, Fudan University, Shanghai, China; ^5^ National Clinical Research Center for Aging and Medicine, Huashan Hospital, Shanghai, China; ^6^ Institute of Neurology, Huashan Hospital, Fudan University, Shanghai, China; ^7^ National Clinical Research Center for Aging and Medicine, Huashan Hospital, Fudan University, Shanghai, China; ^8^ Huashan Hospital, Fudan University, Shanghai, Shanghai, China; ^9^ MOE Frontiers Center for Brain Science, Fudan University, Shanghai, China

## Abstract

**Background:**

Tremendous advancement has been made in biomarker assessment, molecular neuroimaging and disease modifying therapy in neurodegenerative cognitive disorders. China is lacking clinical cohorts that deeply profile patients from memory clinic that integrate both comprehensive clinical assessment and multi‐modal biomarker examinations.

**Methods:**

The Shanghai Memory Study (SMS) is an ongoing prospective hospital‐based cohort study. Participants underwent extensive clinical and neuropsychological assessments, genotyping, multimodal magnetic resonance imaging (MRI), positron emission tomography (PET) imaging, and various biospecimen collection. In this article, the rationale, aims, study design, data collection, and future directions of SMS were summarized. Preliminary analyses including plasma biomarker assay using the Simoa platform, molecular neuroimaging of Aβ PET and tau PET were performed across different diagnostic groups.

**Results:**

A total of 2,001 participants with cognitive complaints were enrolled, including 115 with subjective cognitive decline (SCD), 938 with mild cognitive impairment (MCI), and 948 with dementia. The proportions of individuals with A+/T+ in PET scans were 15.8% in SCD cases, 51.2% in MCI cases, and 100% in AD dementia. And the proportions of individuals with A+/T‐ in PET scans were 5.3% in SCD cases and 3.3% in MCI cases. In the analysis with plasma biomarkers, as the CDR score increased, Aβ40, Aβ42, and Aβ42/Aβ40 ratio exhibited a decreasing trend, whereas t‐tau, NfL, and *p*‐tau181 showed an increasing trend. In a subcohort of 251 patients with amnestic MCI (aMCI), during a median follow‐up of 4.7 years, 88 (35.1%) progressed to AD dementia, 8 developed non‐AD dementia. Individuals with low Aβ42/Aβ40 and high *p*‐tau181 at baseline demonstrated the highest risk of developing AD (hazard ratio = 4.83, 95% CI 2.37–9.86).

**Conclusions:**

By offering an integrated framework for investigating cognitive disorders, SMS will facilitate the exploration of AD pathogenesis and deepen the understanding of cognitive disorders.